# Audit of clinical and laboratory parameters of hemoglobin SS patients in a Nigerian teaching hospital

**DOI:** 10.1080/07853890.2022.2129090

**Published:** 2022-11-13

**Authors:** Titilola Stella Akingbola, Oladapo Wale Aworanti, Sunday Peter Ogundeji

**Affiliations:** Department of Hematology, University of Ibadan/University College Hospital, Ibadan, Nigeria

**Keywords:** Clinical parameters, sickle cell anaemia, steady state, vaso-occlusive crisis, hematology

## Abstract

**Background:**

The burden of Sickle cell anaemia (SCA) is huge in Sub Sahara Africa as it affects 1–2% of the population. HbSS impacts negatively on the quality of life of the sufferers. The clinical manifestations start between 3 and 5 months of life as a result of reduction in foetal hemoglobin.

**Objectives:**

This study describes the clinical and laboratory characteristics of HbSS patients at presentation in steady state, vaso-occlusive and hemolytic crises states.

**Material and method:**

This was a cross sectional, analytical study. Ninety HbSS participants were divided into three groups; steady state, hemolytic and vaso-occlusive crises with 30 individuals in each group. The survey contained sections on bio-data and past medical history obtained from the patients’ notes and results of laboratory tests. Data were analysed using SPSS version 23.0. Results were considered statistically significant if *p* < 0.05.

**Results:**

Ninety participants were analysed in this study. The mean age of the participants was 29.4 ± 8.9 years. Only one-third of the participants were diagnosed within the first year of age. Forty-seven (52.2%) participants have steady state haematocrit in the range of 21–25%. All the participants experienced bone pain in a year, about 25% of these participants had more than three episodes of pain per year. There was a statistically significant difference in the mean values of PCV (*p* < .001), WBC (*p* < .001), platelet (*p* = .008), ANC (*p* < .001), ALC (*p* < .001), AMC (*p* < .001), reticulocyte count and ISC % among the different categories.

**Conclusion:**

This study established the fact that only a minority of the SCD patients are diagnosed in the first year of life and vaso-occlusive crisis is the most frequent reason for hospital presentation. We therefore recommend the institutionalisation by government policy, neonatal screening programme in Nigeria.

**KEY MESSAGES**
- The study highlight delay in early diagnosis of SCA due to unavailability of neonatal diagnosis program in our setting.
- Bone pain remains the major cause of presentation for SCA and most patients presented after a day of onset of pain to the hospital.

## Introduction

Sickle cell anaemia (SCA) is a genetic disorder that results from inheritance of homozygous hemoglobin S from both parents [1–4]. Hemoglobin S is a product of a single nucleotide mutation, this leads to replacement of glutamic acid by valine in the position six of the beta globin chain [1,3]. These changes lead to crystallisation of the soluble hemoglobin and formation of sickled red cells in hypoxic state [2,4].

The sickle cell gene has a worldwide distribution but the impact varies from one part of the world to another. The major impact is in the Sub Saharan Africa (SSA) with the highest in Nigeria with the prevalence of 20–30% and 2–3% for sickle cell trait and HbSS respectively [5,6]. The impact is however lesser in the Middle East, Greece, and aboriginal tribes in India [5]. The high prevalence in the malaria endemic region of the world is attributable to a phenomenon termed balanced polymorphism in which the sickle cell trait confers a selective survival advantage against malaria [7].

The quality of life of patients with HbSS is impaired by recurrent vaso-occlusive and hemolytic crises, which may last a few hours to several weeks, with many requiring hospitalisation and transfusion and attendant financial implications on care-givers [1–3]. The clinical manifestations of this disease start between 3 and 6 months of age, this is as a result of reduction in fetal hemoglobin [1,2,8]. Almost all the body organs and systems are affected by vaso occlusion caused by the sickled red cells with varying severity of the disease based on the level of fetal hemoglobin [1,2,9].

Medical care for sickle cell disease has improved over the years due to better understanding of the pathophysiology of the disease [10]. There is therefore improved survival into adulthood which now engenders a wide spectrum of clinical presentation that needed to be brought to the attention of clinicians and caregivers. This study therefore describes and compares the clinical and laboratory characteristics of different HbSS patients at presentation in steady, vaso-occlusive and hemolytic crises states.

## Methods

This was a descriptive cross-sectional study of 90 adult HbSS patients diagnosed with hemoglobin electrophoresis who are being followed up at the Hematology department of the Teaching Hospital in Nigeria between January and June 2017. They were classified into three groups based on their clinical presentations: steady state, hemolytic and vaso-occlusive crises with 30 individuals in each group. Steady state patients presented for routine follow up visits at the Hematology Out-patient clinic while the other two groups presented in either Hemolytic or Vaso occlusive crisis to either the Hematology Day-care Unit (HDCU) or the emergency department of the hospital. Steady state is defined as stable health state in HbSS patients who did not have bone pain or any other crisis and no blood transfusions in the previous 2 months [10]. Vaso-occlusive crisis (VOC) group: Vaso-occlusive crisis is defined as the occurrence of pain in the extremities, back, chest (ribs, sternum) that lasted for at least 2 h, led to a hematology day-care unit visit, and could not be explained except by sickle cell disease without features of hemolysis [11,12]. Hemolytic crisis is defined as ≥3% reduction in hematocrit, marked reticulocytosis, circulating nucleated red blood cells, polychromasia, unconjugated bilirubinemia and increased urobilinogen in sickle cell anemia [11,12]. The individuals with concurrent overt infection, pregnancy, other SCD, those with concurrent crises and on hydroxyurea (HU) were excluded. Those on Hydroxyurea were excluded due to low penetration of HU in our environment.

All the participants were interviewed by the researcher/attending physicians and questionnaires were completed from information obtained from the participants and case notes. The questionnaire contained sections on bio-data, past medical history, complications of HbSS throughout life and detailed clinical assessment of bone pain and hemolytic features. The complications assessed in the participants included chronic leg ulcer, avascular necrosis, priapism, cholelithiasis, pulmonary hypertension, infertility, stroke, chronic osteomyelitis and pentazocine addiction.

### Assessment of clinical severity

All HbSS patients were assessed for pain, general and organ specific signs and symptoms. Pain assessment included the pain site (ribs, sternum, back, lower or upper limbs), pain duration (up to 3 days; 4–7 days or more than 7 days) and Pain intensity based on single dimensional verbal pain numerical rating system [13]. The pain was categorised as mild for verbal pain numerical score 0–2; as moderate for score of 3–4, severe for score of 5–6, very severe 7–8 and worst possible pain 9–10.

Also, with the aid of the questionnaire, patients that presented with deep amber- coloured urine were assessed for the duration, deepening yellowness of the eyes as confirmed by the attending physician and they were managed based on the clinical presentation with most having to be transfused.

After an informed consent and administration of drugs, Venous blood was collected from all the participants at the time of presentation to the hospital for analysis. Hematological parameters-complete blood count was done using SYSMEX analyser XS 1000i model and peripheral blood film done on a glass slide using May Grunwald Giemsa stain 555 according to manufacturer’s instruction. Bilirubin was estimated using Abott Architect 1000i automated analyser. Urine sample was also taken from the patients for urinalysis using Medi-Test Combi 10; this is to further group the patient into either the hemolytic or vaso-occlusive crisis group. Normal white blood cell range was taken to be 2.5–9.7 × 10^9^/L [14]. Irreversible sickle cell percentage was obtained from the PBF review as the percentage of sickle red cells in ten 100× microscopic field as defined by Ofelia et al. [15]. Ethics review committee of the hospital approved the study (UI/EC/14/0290). All participants gave written informed consent.

### Statistical analysis

Data collected was recorded, validated, and analysed using the IBM Statistical Package for Social Sciences (SPSS) Statistics for Windows, Version 23.0. Armonk, NY: IBM Corp. Data was assessed for normal distribution using Kolmogorov-Smirnov and Shapiro-Wilk statistics. Descriptive and Inferential statistics were applied in the course of the analysis. Univariate statistic for continuous normally distributed data was presented as mean and standard deviation while proportions were used to summarise categorical data. Frequencies were shown in tables. Multivariate analysis for categorical variables was by chi-square and continuous normally distributed variable by independent *t*-test and ANOVA. Results were considered statistically significant if *p* < .05.

## Results

### Socio-demographic

Ninety participants were analysed in this study. The mean age of the participants was 29.4 ± 8.9 years. Majority (32/90, 35.5%) of the participants were between 30 and 39 years while only five participants were aged 50 years and above. There were more females than male (M:F, 1.2:1). There were however no significant differences in the characteristics of these participants in occupation (*p* = .059), and education (*p* = .137) (Table 1).

**Table 1.** Socio-demographic characteristics of participants according to their presentation categories (*N* = 90).

Variable	Steady state (*n* = 30)	Hemolytic crisis (*n* = 30)	Vaso-occlusive crisis (*n* = 30)	*p* Value
Grouped age (in years)				
Less than 20	3 (10.0)	6 (20.0)	5 (16.7)	.183
20–29	10 (33.3)	12 (40.0)	8 (26.7)	
30–39	13 (43.3)	6 (20.0)	13 (43.3)	
40–49	3 (10.0)	5 (16.7)	1 (3.3)	
50 and above	1 (3.3)	1 (3.3)	3 (10.0)	
Sex				
Male	11 (36.7)	16 (53.3)	12 (40.0)	.426
Female	19 (63.3)	14 (46.7)	18 (60.0)	
Occupation				
Student	10 (33.3)	13 (43.3)	9 (30.0)	.059
Professionals	8 (26.7)	4 (13.3)	8 (26.7)	
Artisans	4 (13.3)	3 (10.0)	2 (6.7)	
Petty traders	8 (26.7)	4 (13.3)	8 (26.7)	
Unemployed	0	6 (20.0)	3 (10.0)	
Education				
Nil	0 (0)	1 (3.3)	2 (6.7)	.137
Primary	2 (6.7)	5 (16.7)	1 (3.3)	
Secondary	7 (23.3)	3 (10.0)	8 (26.7)	
Tertiary	21 (70)	21 (70.0)	19 (63.3)	

Chi- square analysis.

**Figure 1.**
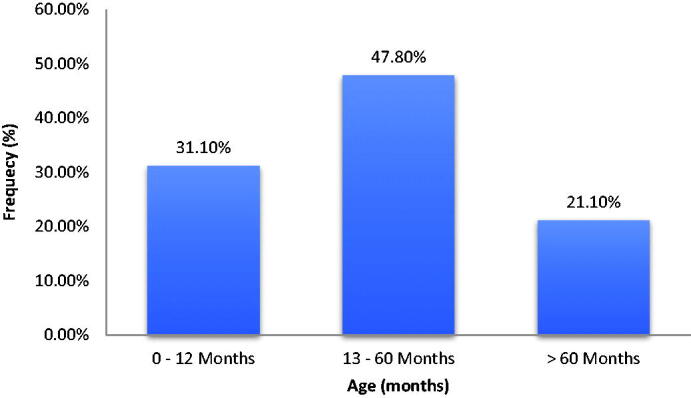
Age at diagnosis of HbSS of the participants.

Only one-thirds of the participants were diagnosed within the first year of age while most of them were diagnosed within their first 5 years of life (Figure 1). One quarter of the participants were not seen in the hospital either for follow up or for management of crisis within 1 year of this study.

**Figure 2.**
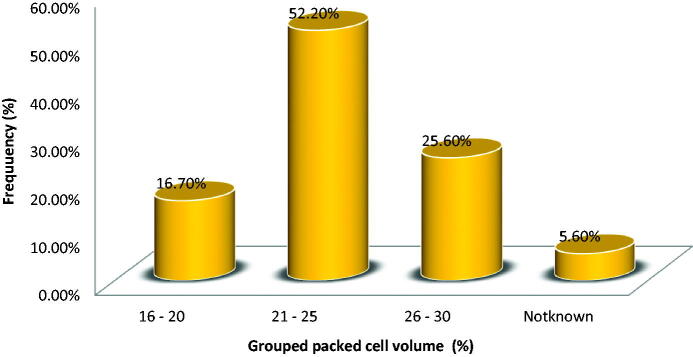
Steady state packed cell volume of participants.

### Blood utilisation and Hospital admission rates

**Figure 3.**
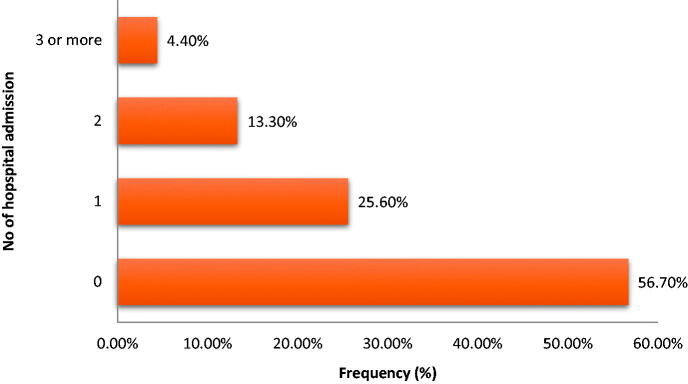
Number of hospital admission in a year in the participants.

Forty-seven (52.2%) of the participants has steady state Hematocrit in the range of 21–25%. However, five (5.6%) of the participants did not know their steady state hematocrit (Figure 2). Also, 19 (about 21%) of the participants had never been transfused and 34 (about 38%) of them had three or more units of blood transfused in their life time. Only less than one-fifth (17.7%) of the participants was admitted at least twice in the last one year prior to enrolment (Figures 3).

**Figure 4.**
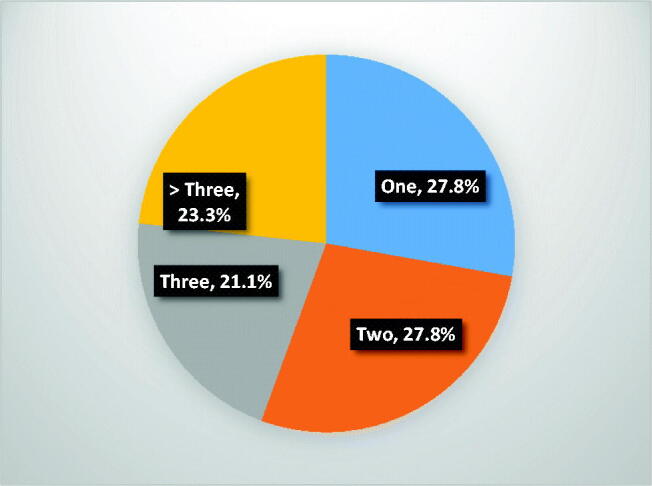
Number of bone pain crisis per year in the participants.

### Frequency of bone pain

All the participants experienced pain episodes in a year, about 25% of these participants have more than three episodes of pain per year while about 30% have only an episode of pain in a year, this is as depicted in the Figure 4.

Among the 30 participants that presented with bone pain crisis, 13 participants (43.3%) presented within 24 h of onset of pain while only one participant (3.3%) presented after 1 week. Also, most of the participants presented with severe bone pain, about 25% of them presented with worst possible pain according to numeric pain score [13] (Table 2).

**Table 2.** Frequency of vaso-occlusive crises in the participants (*N* = 90).

Variables	Frequency	Percentage
Having bone pain now		
Yes	30	33.3
No	60	66.7
Time the pain started (*n* = 30)		
<24 h	13	43.3
2–4 days	12	40.0
4–7 days	4	13.3
More than a week	1	3.3
*Pain score categories (*n* = 30)		
Severe pain	14	46.7
Very severe pain	9	30.0
Worst possible pain	7	23.3

*According to WHO classification of pain.

Figure 4 shows vaso-occlusive crisis in participants with HbSS. Forty (44.4%) of the participants have three or more bone pains per year, while among those participants that presented with bone pain, 43.3% presented within 24 h of bone pain and 46.7% presented with severe pain.

### Frequency of hemolytic crisis

Among the participants, 79 (87.8%) had previous history of passage of deep amber-coloured urine, however from the 30 participants who presented with passage of deep amber-coloured urine, 20 participants (66.7%) presented within 2–4 days of onset of the passage of deep amber-coloured urine while three participants (10%) presented after 1 week. This is shown in Table 3.

**Table 3.** Macroscopic features of hemolysis among respondents (*N* = 90).

Variables	Frequency	Percentage
History of deep amber coloured-urine		
Yes	79	87.8
No	11	12.2
Passing deep amber coloured-urine		
Yes	30	33.3
No	60	66.7
Duration of deep amber coloured-urine (*n* = 30)		
2 − 4 days	20	66.7
4 − 7 days	7	23.3
More than a week	3	10.0
Deepening yellowness of the eyes		
Yes	30	33.3
No	60	66.7

### Hematological parameters

Table 4 shows the hematological parameters of the participants with the VOC group having the highest PCV, with the mean PCV for the group being 24.1 ± 3.6. The hemolytic crisis group had the highest mean white cell count of 16.141 ± 7.330 (×10^3^/µL).

**Table 4.** Hematological parameters of participants (*N* = 90).

Hematological parameters	Steady state (*n* = 30)	Hemolytic crisis (*n* = 30)	Vaso-occlusive crisis(*n* = 30)	**p-Value
PCV (%)	23.2 ± 3.4	18.3 ± 3.9	24.1 ± 3.6	<.001*
WBC (× 10^3^/ µL)	10.704 ± 2.475	16.141 ± 7.330	10.805 ± 3.288	<.001*
Platelet (× 10^3^/ µL)	379.133 ± 120.354	387.300 ± 206.966	354.233 ± 167.427	.008*
ANC (× 10^3^/ µL)	6.746 ± 2.131	9.850 ± 5.106	6.942 ± 2.791	<.001*
ALC (× 10^3^/ µL)	2.659 ± 1.041	4.601 ± 2.837	2.749 ± 1.248	<.001*
AMC (× 10^3^/ µL)	1.017 ± 0.512	1.318 ± 0.809	0.742 ± 0.433	<.001*
MCV (fL)	81.1 ± 6.2	82.5 ± 10.9	76.9 ± 7.8	.220
MCH (pg)	28.0 ± 2.5	28.3 ± 3.6	26.6 ± 2.6	.088
MCHC (g/dL)	34.6 ± 1.8	34.4 ± 1.8	34.4 ± 1.9	.164
Reticulocytes count (%)	10.1 ± 3.8	35.8 ± 10.8	15.3 ± 4.7	<.001*
Irreversible Sickle Cell (%)	4.0 ± 2.2	2.9 ± 1.4	11.24 ± 4.4	<.001*

*Statistically significant **ANOVA was used.

There was a statistically significant difference in the mean values of PCV (*p* < .001), WBC (*p* < .001), platelet (*p* = .008), ANC (*p* < .001), ALC (*p* < .001), AMC (*p* < .001), reticulocyte count and ISC % among the different categories.

### Relevant chemistry

Table 5 depicts the relevant chemistry done on the participants. The total bilirubin of the hemolytic crisis category of the participants is 2.90 ± 2.64 (mg/dL), the highest of all the categories and this was statistically significant.

**Table 5.** Relevant chemistry of the participants (*N* = 90).

Chemical parameters	Steady state (*n* = 30)	Hemolytic crisis (*n* = 30)	Vaso-occlusive crisis (*n* = 30)	**p-Value
pH (urine)	6.65 ± 0.55	6.50 ± 0.54	6.63 ± 0.39	.268
SG (urine)	1.013 ± 0.007	1.013 ± 0.005	1.011 ± 0.005	.145
Total bilirubin (mg/dL)	1.30 ± 1.16	2.90 ± 2.64	1.46 ± 1.31	.001*
Conjugated bilirubin (mg/dL)	0.73 ± 0.680	1.18 ± 1.215	0.85 ± 0.764	.303

*Statistically significant **ANOVA was used.

From the urinalysis, increased urobilinogen is seen only in patients with haemolytic crisis.

### Complications of the disease

**Figure 5.**
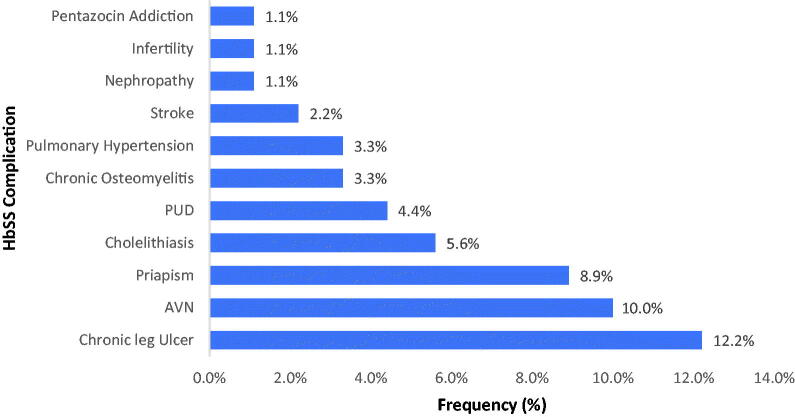
Complications of sickle cell anaemia in the participants.

Chronic leg ulcer is the most common complication seen in the participants, it is seen in 11 participants (12.2%), this is closely followed by Avascular Necrosis of the head of femur, this is seen in nine participants (10%). Priapism is seen in about one in five male participants. This is as shown in Figure 5.

## Discussion

Early diagnosis of HbSS has been advocated as one of the methods to improve survival [16] and reduce morbidity and mortality, however this study revealed that only one-thirds of the participants were diagnosed within the first year of life while four-fifths of the participants were diagnosed within first 5 years of life. Some of the reasons for this delay are low uptake of national newborn screening program and high cost of the procedures involved [17]. This finding is similar to previous studies carried out by Akodu et al. and Brown et al. where most patients were diagnosed within the first 5 years of life [18,19]. Therefore the government must intensive efforts for effective newborn screening program.

The most frequent clinical presentation of HbSS participants in this study was bone pain crisis with about two fifths having three or more bone pain crisis per year and they presented within 24 h. Bone pain crisis is the most consistent and characteristic manifestation of HbSS and the indication for most hospital visits [20]. This is similar to previous work carried out by Adewoyin et al. in which 30% of their respondents have three or more bone pains per year [21].

Another finding in this study is that most of the HbSS participants had previous history of blood transfusion (79%) however, the transfusion requirement is generally low as only 38% have had at least 3 units of blood. These findings are consistent with previous work done by Otaigbe in 2013, that showed less multiple transfusions, though the patients studied were of paediatric age group [22] but in contrast to Akodu et al. who reported a higher prevalence (53%) of recurrent blood transfusion. The reason for this low blood transfusion need may be because these patients were on regular clinic visits where compliance with routine folic acid supplementation was monitored.

The steady state hematocrit ranged between 21% and 25% in almost half of the respondents. The findings in our study is similar to a retrospective review of adults SCA in Benin, Nigeria where the mean hematocrit for sickle cell disease patient in steady state is found to range between 21% and 30% [21].

Our study also showed that white blood cell (wbc) was higher than normal reference range for this environment (ref 2.5–9.7) and the wbc was significantly higher in haemolytic crisis than in steady state and vaso-occlusive crisis (*p* < .05). This finding is consistent with the findings of Gonclaves et al. and Kheikhaei et al., which collectively agreed that SCD is a chronic inflammatory disease [23,24]. It is suggested that increased haematopoietic activity, in addition to transition of the marginating leucocytes to the circulating pool just before onset of or during vaso-occlusive crisis could be responsible for the more elevated level of leukocytosis noted in the three groups [16]. Leukocytosis contribute to the pathophysiology of HbSS by release of microparticles and phophastidyl serine exposure as elucidiated by Picci et al. [25].

As the survival improves, sickle cell disease patients are predisposed to different complications of the disease such as chronic leg ulcer, priapism, chronic osteomyelitis, acute chest syndrome, stroke, pentazocine addiction. In these participants, the commonest complication reported was chronic leg ulcer, this is seen in about 12% of the total participants. The cause of leg ulcer is the vaso-occlusion of skin microvasculature, worsened by trauma, infection, warm climate, and iron overload. This is consistent with the findings of Bazuaye et al. where the incidence of chronic leg ulcer was about 10% [26]. Next to the chronic leg ulcer is priapism, seen in about a quarter of male respondents. Previous findings showed prevalence of priapism to be about 45% in SCD male patients [27], our finding in this study is relatively lower compared to the findings by Nwogoh et al. [28]. The least complications seen were pentazocine addiction, sickle cell nephropathy and infertility.

The influence of genetic modifiers particularly haplotypes and hemoglobin F was not assessed and a relatively small population of HbSS patients was explored in this study; these are some of the limitations of this study.

## Conclusion

This study establishes the fact the only a minority of the SCD patients are diagnosed in the first year of life. This practice has to change so that childhood mortality associated with SCD could be reversed, when early medical interventions are instituted. Furthermore, we also confirmed that the most frequent reason why SCD present to the hospital is vaso-occlusive crisis and this fact opens further vista for translational research into prevention strategies that will be affordable to patients in low-income countries. We therefore recommend the institutionalisation by government policy, neonatal screening program in Nigeria.

## Data sharing policy

The data that support the findings of this study are available on request from the corresponding author, [AOW]. The data are not publicly available due to [restrictions e.g. their containing information that could compromise the privacy of research participants].
